# Supplementary figures and images for: In vitro proliferation of Mytilus edulis male germ cell progenitors
Source: PLoS One. 2024 Feb 9;19(2):e0292205. doi: 10.1371/journal.pone.0292205 (PMC10857695; doi:10.1371/journal.pone.0292205)

A

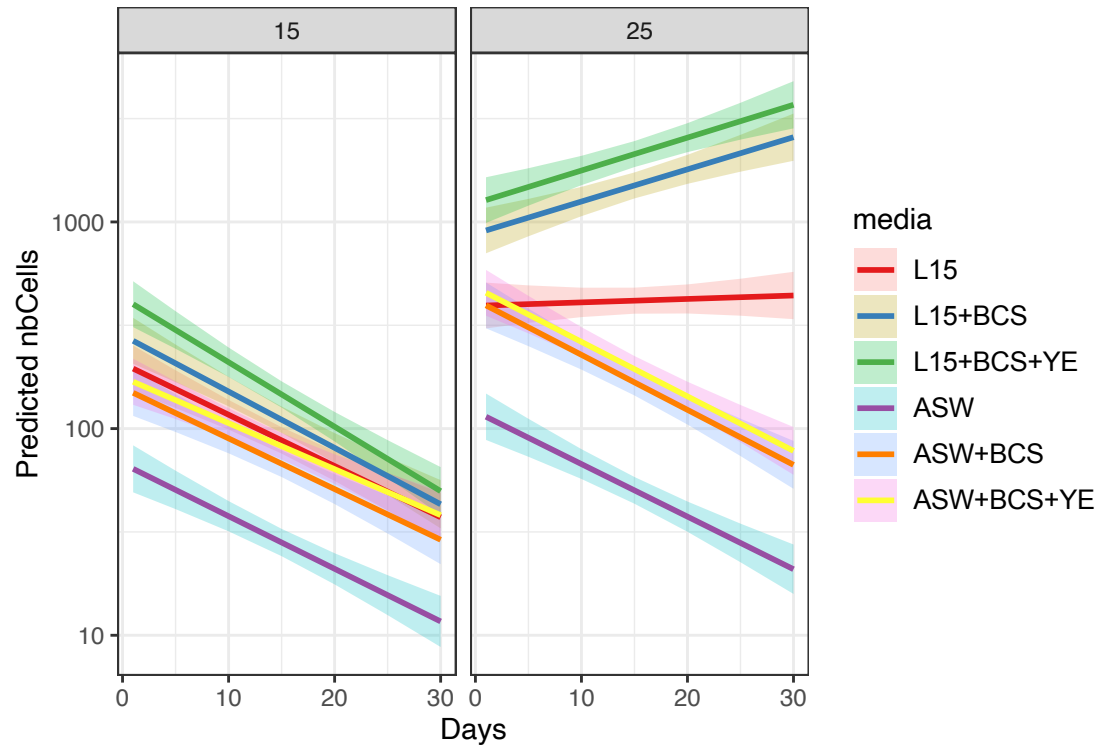

B

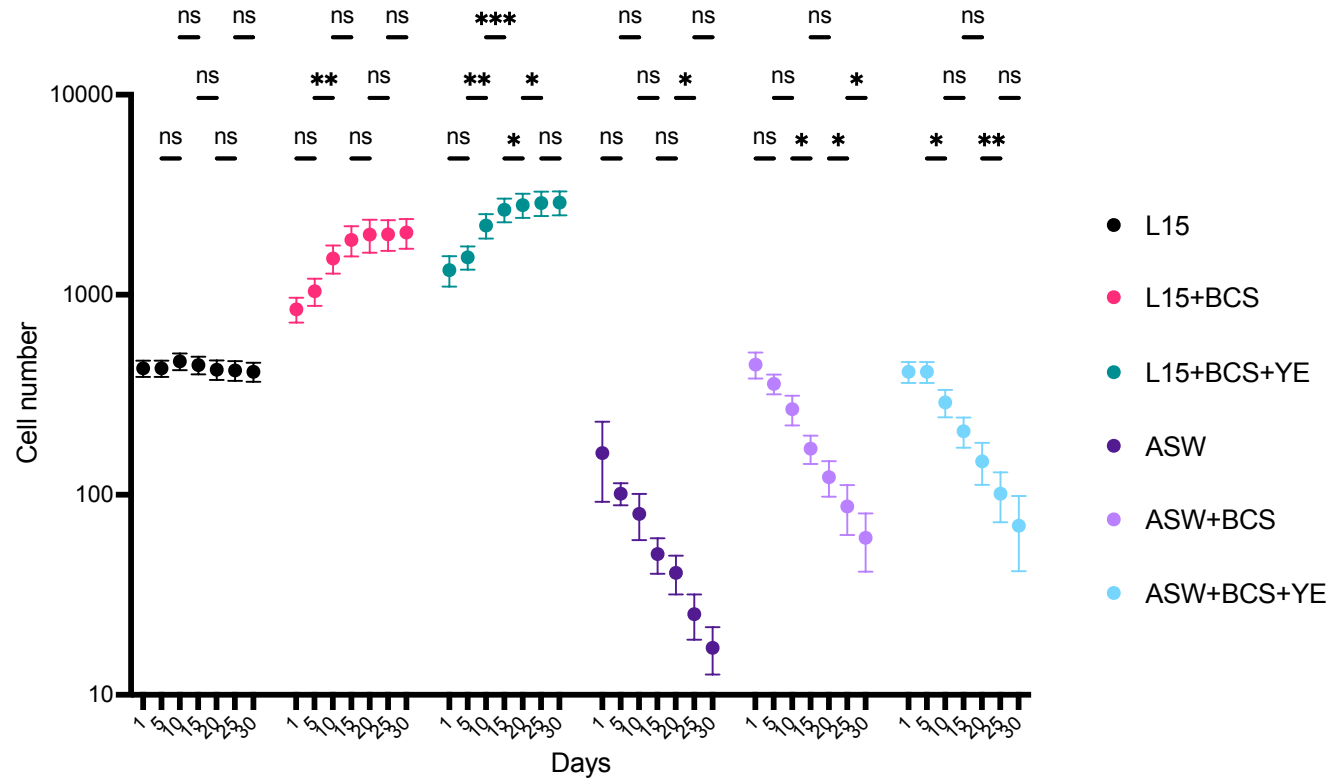

Supplement: S1 Fig — A) GLM model with a confidence interval for cell number in different media. There is no overlap of the confidence intervals at 25°C, showing that cell growth is positive and significantly different in L15_BCS and L15+BCS+YE compared to L15 alone. Growth is negative in all other tested conditions. B) Tukey’s comparison of mean cell number at different times for all media tested at 25°C. Significance is shown only for consecutive measures. ns non significant, *p < 0.05, **p < 0.01, ***p<0.001. (PDF) [file pone.0292205.s001.pdf]

A

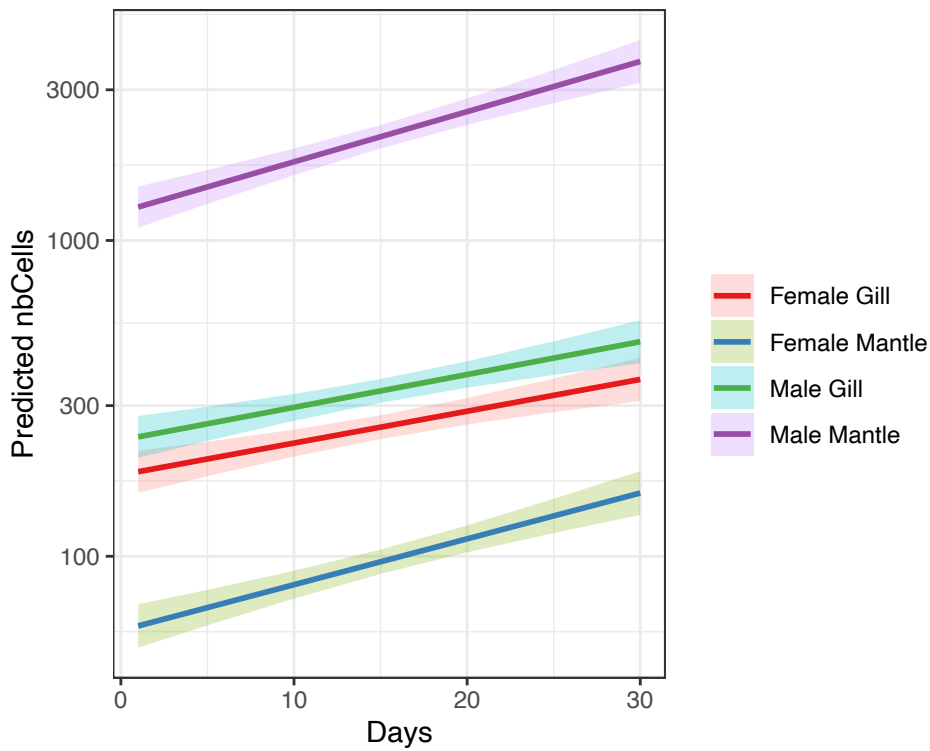

B

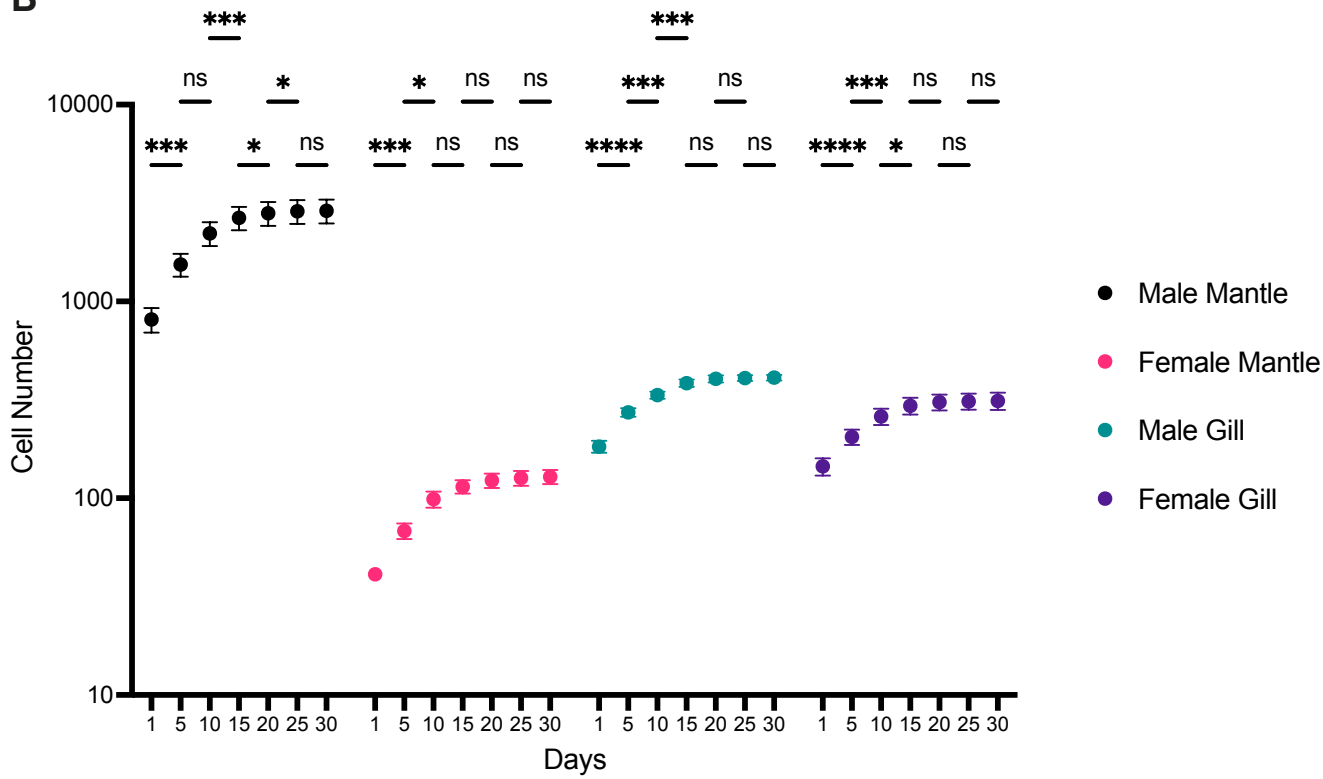

Supplement: S2 Fig — A) GLM model with a confidence interval for cell number and growth obtained from different tissues. Cells from male and female mantle increased more over time than cells from male and female gills. There is no overlap in the models, meaning that all cultures behaved differently. B) Tukey’s comparison of mean cell number at different times for all tissues tested. Significance is shown only for consecutive measures. ns non significant, *p < 0.05, **p < 0.01, ***p<0.001. (PDF) [file pone.0292205.s002.pdf]
