# Supplementary material for: In vitro proliferation of Mytilus edulis male germ cell progenitors
Source: PLoS One. 2024 Feb 9;19(2):e0292205. doi: 10.1371/journal.pone.0292205 (PMC10857695; doi:10.1371/journal.pone.0292205)
Supplement: Attachment — Submitted filename: In vitro proliferation of Mytilus edulis male germ cell progenitors.docx [file pone.0292205.s003.docx]

**Title:** *In vitro* proliferation of *Mytilus edulis* male germ cell progenitors

**Proliferation:** Only markers have been used to identify germ cell progenitors. Whether any sub-cultures could be done is not mentioned. No experiments to measure actual proliferation (like BrdU assay) is done. BrdU staining or assay should be done during different days of incubation to show that cells are actually proliferating in vitro. Mere presence of germ line markers cannot verify if actual proliferation is happening or not. So, according to me, manuscript does not do justice to the title.

**Inconsistency:** Inconsistency in methodology and results- Only 2 temperature mentioned in methodology, but results talk about 4 temperatures tested, but then goes on to elaborate only about two specific temperatures 15 and 25 ^o^C. Methodology does not describe the three cell dissociation methods tested, but only the final chosen method is described.

**Significant difference obtained or not?**

(a) In the figure 1B and C, significant differences between days is not mentioned. A graph with mean and standard deviation, and significance values denoted as letters will be easier to interpret. The current graphs do not show any symbols to indicate if there were significant differences, and the graphs show overlapping values between the different days of observation (see figure 1C), creating doubt if there was actually any significant difference (i.e. if there was actually any significant cell proliferation). The authors say that cell proliferation (male mantle) occurred only until Day 15 like has been previously reported by Attaallah et al. 2020. In that case, what new findings do authors have to contribute?
(b) Fig 2 A and B: In the study based on cell proliferation rate in different sex, the results say that female mantle cells also proliferated till Day 15. So, what is the difference in cell proliferation rate between male and female mantle cells? On what terms do you say that male mantle cells are better? Also, significant differences are not plotted on the graph. Also, many values in female mantle cells overlap with that of male mantle cells. The graph will be clearer with mean and standard deviation plots. You can keep either figure 2A or figure 2B. Two graphs convey more or less the same matter.
(c) **Immunofluorescence:** Fluorescence measured on 1, 5 and 25. It is said that proportion of germ cells increased on Day 25, whereas cell counting experiment says cell proliferation occurred only until Day 15. These may be confounding results. It may not be termed proliferation, but just a shift in cell population. So, this statement is inappropriate “DDX4-positive cells were the only cells that significantly proliferated in culture, and only from male tissue samples”. Keep either Figure 5 or 6. Two graphs convey more or less the same matter. Again, letters to denote significant differences among groups are required.

**Antibiotics:** Wide literature including personal experience shows that Amphotericin B is toxic to cells in vitro. How could you manage a long-term cell culture with Amphotericin B?

**Figure 3** is difficult to interpret using the arrowhead legends. Example, Type A cells are supposed to exhibit both antigens, but I cannot see both antigens in the representative picture they have shown in Figure 3 (Panel A). May be both antigens are present on the cells, but the picture is not clear. Similarly for other images.

**Figure 7** is a beautiful depiction of cell differentiation in vitro

**Overall comment:** the effort is worth appreciating but the results seem to be confounding, and the title and conclusions are misleading.
